# (2*R*,3*S*)-Dihydroxybutanoic Acid Synthesis as a Novel Metabolic Function of Mutant Isocitrate Dehydrogenase 1 and 2 in Acute Myeloid Leukemia

**DOI:** 10.3390/cancers12102842

**Published:** 2020-10-01

**Authors:** Jeffrey R. Idle, Katja Seipel, Ulrike Bacher, Thomas Pabst, Diren Beyoğlu

**Affiliations:** 1 Arthur G. Zupko’s Systems Pharmacology and Pharmacogenomics, Arnold and Marie Schwartz College of Pharmacy and Health Sciences, Long Island University, Brooklyn, NY 11201-5423, USA; jeff.idle@liu.edu; 2 Department of BioMedical Research, University of Bern, 3008 Bern, Switzerland; katja.seipel@dbmr.unibe.ch (K.S.); thomas.pabst@insel.ch (T.P.); 3 Department of Hematology, Inselspital, Bern University Hospital, University of Bern, 3010 Bern, Switzerland; veraulrike.bacher@insel.ch; 4 Department of Medical Oncology, Inselspital, Bern University Hospital, University of Bern, 3010 Bern, Switzerland

**Keywords:** acute myeloid leukemia, isocitrate dehydrogenase 1, isocitrate dehydrogenase 2, metabolomics, biomarker, (2*R*)-hydroxyglutaric acid, (2*R*,3*S*)-dihydroxybutanoic acid, oncometabolite

## Abstract

**Simple Summary:**

Acute myeloid leukemia (AML) is one of several cancers where cancer proliferation occurs under the influence of an aberrant metabolite known as an oncometabolite produced by a mutated enzyme in the cancer cell. In AML, mutant isocitrate dehydrogenases produce the oncometabolite 2-hydroxyglutarate. We screened AML patients with and without mutant isocitrate dehydrogenases by using a technique known as metabolomics, which measures many different metabolites in patient plasma. It was observed that another metabolite, 2,3-dihydroxybutyrate, was produced in larger amounts in patients with mutated isocitrate dehydrogenase and correlated strongly with 2-hydroxyglutarate levels. Moreover, 2,3-dihydroxybutyrate was a better indicator of the presence of mutated isocitrate dehydrogenase in the cancer than the known oncometabolite 2-hydroxyglutarate. These findings may lead to the characterization of 2,3-dihydroxybutyrate as a novel oncometabolite in AML, which would bring a fuller understanding of the etiology of this disease and offer opportunities for the development of novel therapeutic agents.

**Abstract:**

Acute myeloid leukemia (AML) frequently harbors mutations in isocitrate 1 (*IDH1*) and 2 (*IDH2*) genes, leading to the formation of the oncometabolite (2*R*)-hydroxyglutaric acid (2R-HG) with epigenetic consequences for AML proliferation and differentiation. To investigate if broad metabolic aberrations may result from *IDH1* and *IDH2* mutations in AML, plasma metabolomics was conducted by gas chromatography–mass spectrometry (GC–MS) on 51 AML patients, 29 *IDH1*/*2* wild-type (WT), 9 with IDH1R132, 12 with IDH2R140 and one with IDH2R172 mutations. Distinct metabolic differences were observed between IDH1/2 WT, *IDH1R132* and *IDH2R140* patients that comprised 22 plasma metabolites that were mainly amino acids. Only two plasma metabolites were statistically significantly different (*p <* 0.0001) between both IDH1R132 and WT *IDH1/2* and IDH2R140 and WT IDH1/2, specifically (2*R*)-hydroxyglutaric acid (2R-HG) and the threonine metabolite (2*R*,3*S*)-dihydroxybutanoic acid (2,3-DHBA). Moreover, 2R-HG correlated strongly (*p <* 0.0001) with 2,3-DHBA in plasma. One WT patient was discovered to have a D-2-hydroxyglutarate dehydrogenase (*D2HGDH*) A426T inactivating mutation but this had little influence on 2R-HG and 2,3-DHBA plasma concentrations. Expression of transporter genes *SLC16A1* and *SLC16A3* displayed a weak correlation with 2R-HG but not 2,3-DHBA plasma concentrations. Receiver operating characteristic (ROC) analysis demonstrated that 2,3-DHBA was a better biomarker for IDH mutation than 2R-HG (Area under the curve (AUC) 0.861; *p <* 0.0001; 80% specificity; 87.3% sensitivity). It was concluded that 2,3-DHBA and 2R-HG are both formed by mutant IDH1R132, IDH2R140 and IDH2R172, suggesting a potential role of 2,3-DHBA in AML pathogenesis.

## 1. Introduction

Almost a century ago, Otto Warburg described experiments that established the first metabolic anomaly in tumors that was distinct from cells in normal tissues, a preference for the fermentation of glucose to lactate by aerobic glycolysis [1,2], subsequently to become known as the Warburg effect [3]. With the characterization of nucleic acids in the 1950s and the subsequent discovery of oncogenes and tumor suppressor genes, the role of aerobic glycolysis might play in oncogenesis began to draw less attention. Furthermore, aerobic glycolysis was viewed as an inefficient means of ATP generation compared to mitochondrial oxidation and therefore the advantage of glycolytic metabolism to the tumor was unclear. Nevertheless, tumor glycolysis can occur independently of oxygen and produce ATP at a rate 10–100 times faster than that by mitochondrial oxidation. However, the recognition that cancer cell metabolism is tailored to enable the conversion of nutrients into the lipids, amino acids and nucleotides needed for the generation of a new cell, moved the focus from efficiency of ATP production to the metabolism of nutrients into cellular building blocks. It was recognized that proliferating cells must switch from carbon catabolism for ATP production to the synthesis of the macromolecular precursors acetyl-CoA, non-essential amino acids, and ribose [3]. In addition, the uncovering of the mechanism by which the Krebs cycle enzymes fumarate hydratase and succinate dehydrogenase could function as tumor suppressors [4] helped place energy metabolism at the center of cancer biology. Given the foregoing, it is surprising that relatively few endogenous metabolic pathways have been recognized as essential elements of oncogenesis. Nevertheless, reprogramming of energy metabolism has been added to the hallmarks of cancer [5]. However, it would be work on two other Krebs cycle intermediates, isocitrate and 2-oxoglutarate (2-OG), in particular in glioma and acute myeloid leukemia (AML), that would lead to the concept of an “oncometabolite”.

A genome-wide analysis of gliomas revealed mutations in the isocitrate dehydrogenase genes *IDH1* and *IDH2* that diminished the NADP-dependent conversion of isocitrate to 2-OG when expressed in cell culture [6]. Mutant *IDH1* and *IDH2* also occur in AML, which is invariably heterozygous for these *IDH* mutations and so retains functional isocitrate metabolism [7]. It has been stated that *IDH1* and *IDH2* mutations are mutually exclusive in AML [8,9,10]. However, this does not preclude the occurrence of rare cases bearing both mutations detected in particular with the use of next-generation sequencing [11]. The mutant neomorphic IDH enzyme, in contrast to the wild-type (WT) enzyme, converts 2-OG to (2*R*)-hydroxyglutaric acid (2R-HG; D-2-hydroxyglutaric acid) that can accumulate to 10 mM concentration or greater [12] and was thought to contribute to leukemogenesis [7,13]. Subsequently, it was reported that 2R-HG producing cells displayed global DNA hypermethylation, a specific hypermethylation signature, harbored impaired DNA demethylase TET2 catalytic function [14], with inhibition of 2-OG-dependent chromatin-modifying enzymes, including JmjC histone lysine demethylases [15]. As a consequence, these effects of 2R-HG that alter the epigenetic landscape of both AML [16] and of glioma [17] resulted in 2R-HG being labeled as an oncometabolite [7].

Metabolomics is a powerful tool for the examination of cancer metabolism [18]. We have recently reviewed the history of metabolomics and its potential for uncovering disease biomarkers [19,20]. Using three separate mass spectrometry-based lipidomic platforms, we have reported a detailed metabolomic investigation of the plasma lipid profile of AML [21]. We found a decline in sphingolipids, phosphocholines, triglycerides and cholesterol esters probably due to enhanced fatty acid oxidation in AML cells. Arachidonic acid and its precursors were elevated in AML, predominantly in patients with a high number of blasts in bone marrow or blood and an adverse prognostic risk. PGF2α was also elevated but in patients with low blast counts in bone marrow or blood and with a favorable prognostic risk. A wide range of individual lipids were therefore altered in AML patient plasma compared to healthy controls. A number of metabolomic and lipidomic investigations in AML have been reported [22,23,24,25,26,27,28,29,30] and these have been recently reviewed [31]. None of these studies addressed the changes to the plasma metabolome conferred by mutated *IDH1* and *IDH2*. It is understood that such mutations result in production of the oncometabolite 2R-HG. However, what is not known is what additional primary or secondary metabolic changes are a consequence of *IDH* gene mutation and may contribute to the pathogenesis of AML and/or its resistance to chemotherapy. 

The purpose of this study was to define metabolic changes visible in the plasma of AML patients as a result of *IDH* gene mutation. Towards this aim, we have conducted an investigation of AML patients with and without mutations at IDH1R132, IDH2R140 and IDH2R172 using gas chromatography–mass spectrometry (GC–MS) metabolomics with multivariate and univariate data analysis. It is concluded that mutations at IDH1R132 and IDH2R140 have differential effects on the plasma metabolome and that both 2R-HG and the threonine metabolite (2*R*,3*S*)-dihydroxybutanoic acid (2,3-DHBA; 4-deoxthreonic acid) were elevated in plasma as a result of *IDH1* or *IDH2* mutation. Furthermore, these two metabolites were highly correlated, strongly suggesting that not only 2R-HG but also 2,3-DHBA is produced by mutant IDH.

## 2. Results

### 2.1. Metabolomic Analysis 

Plasma samples were analyzed in triplicate by GC–MS and a total of 67 chromatographic peaks were identified using the NIST 14 spectral library and an in-house library of 120 authentic standards. Six metabolites each appeared as two peaks due to multiple derivatives and these concentrations were summed to yield relative concentrations for 60 identified metabolites that were subjected to multivariate data analysis using orthogonal projection to latent structures-discriminant analysis (OPLS-DA) [32]. 

#### 2.1.1. Comparison of AML Patients with WT IDH1/2, Mutated IDH1 and Mutated IDH2

**Figure 1:**
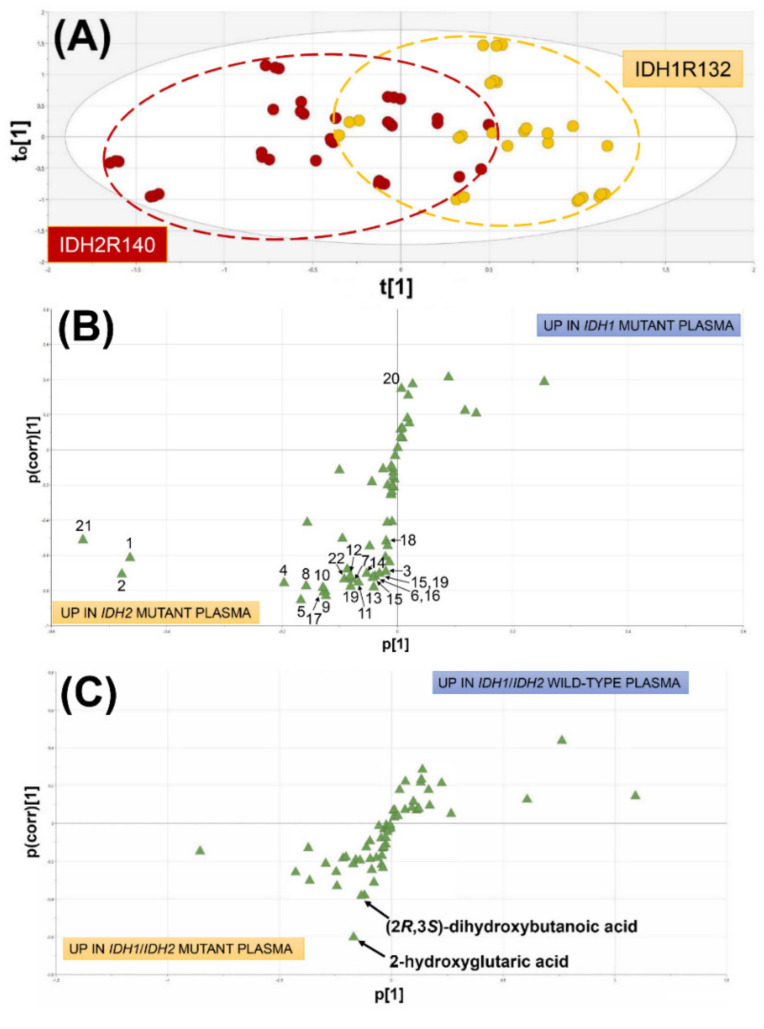
Plasma metabolomic analysis of acute myeloid leukemia (AML) patients with IDH1R132 versus IDHR140 mutations and wild-type (WT) *IDH1/2* patients versus mutated *IDH1/2*. (**A**) Orthogonal projection to latent structures-discriminant analysis (OPLS-DA) scores plot for IDH1R132 (yellow) versus IDHR140 (burgundy) showing clustering and partial separation of the two metabolic phenotypes. t[1] and t_0_[1] are the first principal component and the first orthogonal component, respectively. (**B**) OPLS-DA loadings S-plot showing the up- and down-regulated plasma metabolites responsible for the separation in scores in (**A**). Upper right and lower left quadrants represent the metabolites that are upregulated in *IDH1* mutant plasma and *IDH2* mutant plasma, respectively. Metabolites labeled 1 to 22 correspond to those listed in Table 1. p[1] and p(corr)[1] are related to relative concentration and correlation to the model, respectively. (**C**) OPLS-DA loadings S-plot showing the upregulated plasma metabolites in WT *IDH1*/*IDH2* (upper right quadrant) and mutant *IDH1*/*IDH2* (lower left quadrant) AML patients. Note that only two statistically significantly different metabolites were found between WT and mutant AML plasmas, 2R-HG, as expected, and the unexpected finding of 2,3-DHBA.

Metabolite plasma concentrations were compared for nine patients with IDH1R132 mutations and 12 with IDH2R140 mutations using OPLS-DA. Figure 1A shows the OPLS-DA scores plot for this comparison. Two metabolic phenotypes are visible corresponding to IDH1R132 and IDH2R140. It is clear that the mutation of *IDH2* had a more profound effect on cellular metabolism than the mutation of *IDH1*, with 21 elevated plasma metabolites compared to only one elevated metabolite for *IDH1* mutation. Table 1 itemizes the metabolic differences between WT and mutations of *IDH1* and *IDH2*.

Interestingly, 14 α-amino acids were varied highly statistically significantly in these three IDH genotypes. The general pattern of amino acid changes was that they were lower in IDH1R132 than IDH1/2 wild types but higher in IDH2R140 than wild types. The same pattern was found with all the other metabolites listed in Table 1, except hexanoic acid. The OPLS-DA loadings S-plot (Figure 1B) shows the relative abundance of the plasma metabolites as measured on the abscissa by p[1] that indicates that urea (#21), mannose (#2) and galactose (#3) were the most abundant plasma metabolites resulting from *IDH2* mutation. In contrast, hexanoic acid associated with *IDH1* mutation appeared to be a relatively minor metabolite based upon its p[1] value (Figure 1B) and its relative concentration in Table 1. The relative abundance of the α-amino acids in IDH2R140 plasma is unrelated to their natural abundance genome wide in human proteins, where leucine has the highest abundance (9.97%) and tryptophan the lowest (1.22%) [34]. It is unlikely therefore that these α-amino acids derive from nonspecific proteolysis. However, an altered plasma profile of free amino acids in AML has been previously reported, with elevated glutamate, tryptophan, ornithine, and glycine relative to the plasma of healthy control subjects. In addition, plasma serine, methionine, and taurine concentrations were lower in AML than in control plasma [35]. This same group reported elevated plasma glutamate and tryptophan in both lung and breast cancer patients [36]. Unlike the previous report [35], plasma samples in the current investigation were not collected under fasting conditions. 

#### 2.1.2. Comparison of AML Patients with Wild-Type IDH1/2 and Mutated IDH1/IDH2 

**Figure 2:**
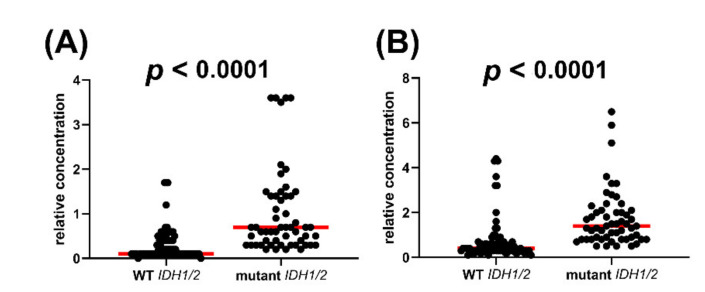
Plasma concentrations of (**A**) (2*R*)-hydroxyglutaric acid and (**B**) (2*R*,3*S*)-dihydroxybutanoic acid in patients with WT and mutant *IDH1/2*. Red horizontal lines represent median values and distributions were compared using the nonparametric Mann–Whitney test. Note the similar distribution of these two metabolites and the presence of outliers in the *IDH1/2* WT genotypes, suggesting the presence of mutations additional to IDH1R132 and IDH2R140 that were not determined.

Metabolomic analysis was conducted on 29 AML patients with WT IDH1/2 compared to 21 AML patients with IDH1R132 and IDH2R140 mutations. The OPLS-DA loadings S-plot is shown in Figure 1C. No plasma metabolites were statistically significantly elevated in WT patients but those bearing IDH1R132 or IDH2R140 mutations had two statistically significantly elevated plasma metabolites, the expected 2R-HG (*p <* 0.0001) and the unexpected finding of 2,3-DHBA (*p <* 0.0001). Figure 2 shows the distribution of relative concentration in plasma for both these plasma metabolites in WT *IDH1/2* AML patients and mutant *IDH1/2* patients. 

**Figure 3:**
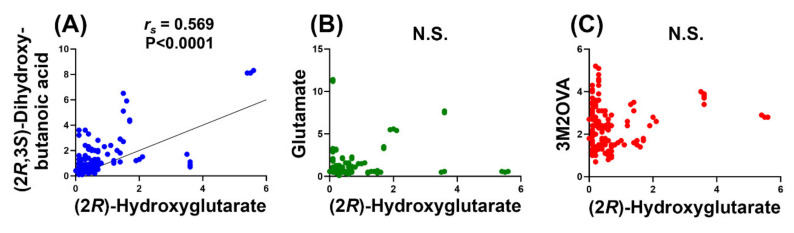
Correlations between (2*R*)-hydroxyglutarate and (**A**) (2*R*,3*S*)-dihydroxybutanoic acid, (**B**) glutamate, and (**C**) (3*S*)-methyl-2-oxovaleric acid. Correlation analysis was conducted using the nonparametric Spearman rank correlation, which was highly significant for (2*R*)-hydroxyglutarate and (2*R*,3*S*)-dihydroxybutanoic acid (*r_s_* = 0.569; *p* < 0.0001) but not significant (N.S.) for either glutamate or (3*S*)-methyl-2-oxovaleric acid.

To investigate the potential relationship between 2R-HG and 2,3-DHBA, correlation between these two metabolites was conducted and, as negative controls, between 2R-HG and glutamate and 2R-HG and (3*S*)-methyl-2-oxovaleric acid (3M2OVA) (Figure 3). 

**Figure 4:**
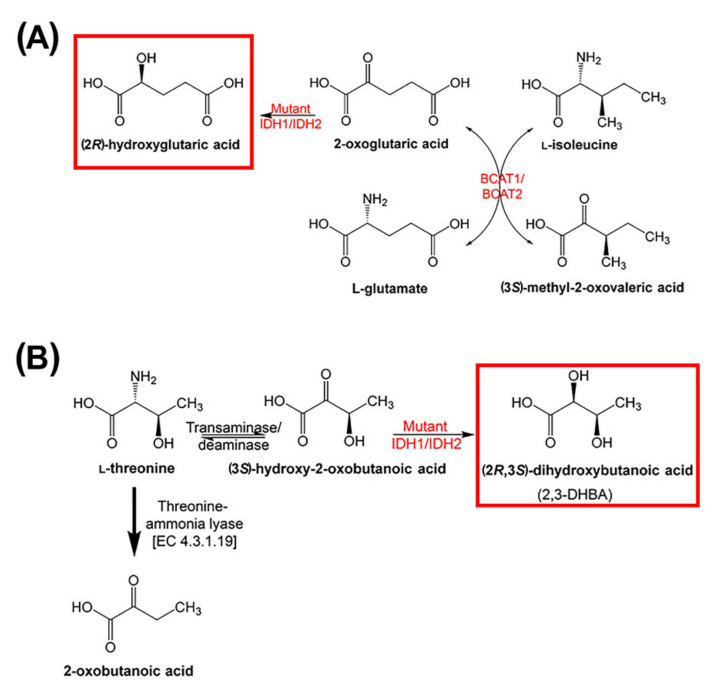
Metabolic pathways showing the formation of (**A**) 2R-HG from 2-OG by mutant IDH1 and IDH2 with the transamination of 2-OG with isoleucine by BCAT1 and BCAT2 to glutamate and 2M3OVA. (**B**) The transamination and reduction of threonine to 2,3-DHBA with the participation of 2,3-DHBA.

The two negative controls were chosen because the 2R-HG precursor 2-OG undergoes transamination with the branched-chain amino acids valine, leucine, and isoleucine mediated by branched-chain amino acid transaminase 1 and 2 (BCAT1 and BCAT2). The transaminase reaction between 2-OG and isoleucine produces glutamate and 3M2OVA (α-keto-β-methylvalerate; KMV) [37] (see Figure 4). In an apparently unrelated pathway, threonine undergoes transamination/deamination to (3*S*)-hydroxy-2-oxobutanoic acid, which is further reduced stereospecifically to 2,3-DHBA (also known as 4-deoxythreonic acid) in an uncharacterized reaction [38,39]. Nuclear magnetic resonance studies also showed that the (2*R*,3*R*)-diastereomer (4-deoxyerythronic acid) was not present in normal human urine [39]. An earlier report stated that 4-deoxythreonic acid was present in large amounts in human urine compared to 4-deoxyerythronic acid [40]. 

The catabolism of threonine is generally considered to proceed by threonine-ammonia lyase [EC 4.3.1.19] to 2-oxobutanoic acid (α-ketobutyrate) (Figure 4B) [41], which can be further utilized to synthesize isoleucine [42]. However, threonine can undergo transamination [41], which produces (3*S*)-hydroxy-2-oxobutanoic acid (α-keto-β-hydroxybutyrate; Figure 4B) [39]. Additionally, studies with [^13^C] threonine in the dog established that 2,3-DHBA derived from threonine [38]. The data and analyses presented here indicate that the final step in the synthesis of 2,3-DHBA is conducted by mutant IDH1 and/or IDH2 in parallel to the synthesis of 2R-HG.

#### 2.1.3. Comparison of WT vs. IDH1R132 and WT vs. IDH2R140 and the Effect of D2HGDH Polymorphism on 2R-HG and 2,3-DHBA Plasma Levels

**Figure 5:**
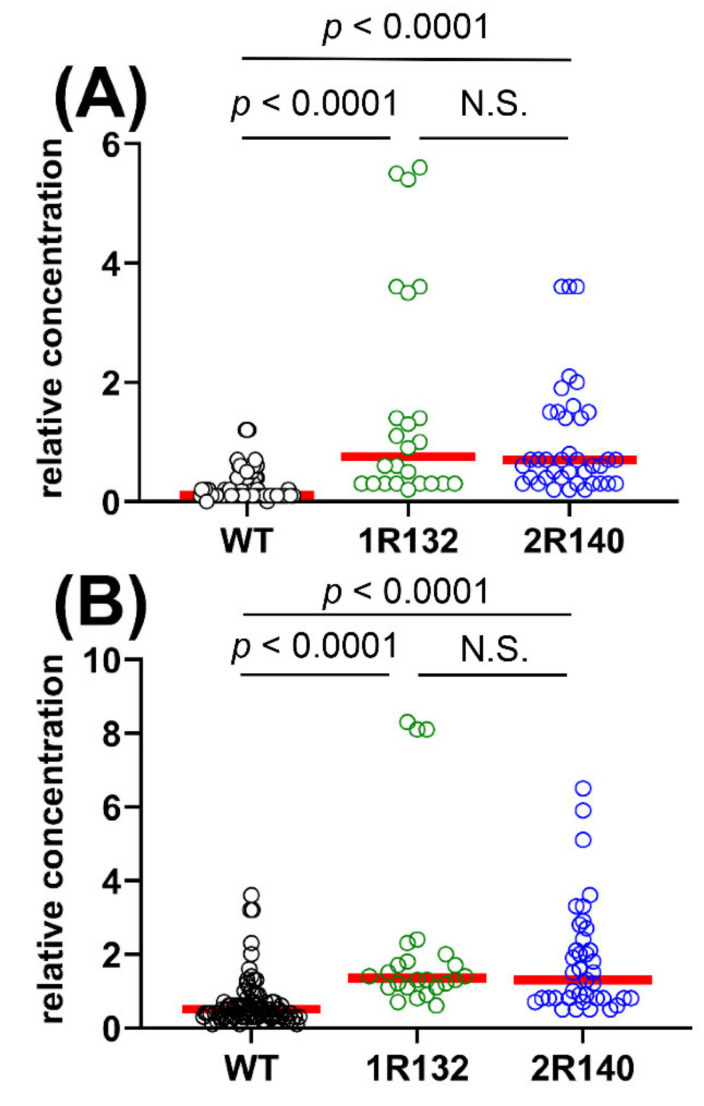
Statistical analysis of (**A**) 2R-HG and (**B**) 2,3-DHBA plasma concentrations in WT vs. IDH1R132 and WT vs. IDH2R140 AML patients. Both comparisons showed statistically significant differences (*p* < 0.0001) by the nonparametric Mann–Whitney test, while plasma concentrations in IDH1R132 and IDH2R140 patients were not statistically significant (*p* > 0.7). Red horizontal lines represent median values in each group. All patients are represented by triplicate plasma analyses by GC–MS.

Figure 2 shows that AML patients with IDH1/2 mutations had highly statistically significantly elevated plasma concentrations of both 2R-HG and 2,3-DHBA. However, it was notable that certain patients with WT IDH1/2 displayed high plasma concentrations of both 2R-HG (Figure 2A) and 2,3-DHBA (Figure 2B). In order to establish if these high plasma levels derived from IDH1 or IDH2, we conducted a separate analysis of WT vs. IDH1R132 and IDH2R140 (Figure 5). During this analysis, we noted that the single IDH2R172 patient had a higher plasma concentration of both 2R-HG and 2,3-DHBA than any IDH1/2 WT patient and the vast majority of the IDH1R132 and IDH2R140 patients (see Table S2). 2R-HG plasma concentrations were higher for the IDH2R172 patient than the IDH1/2 WT patients (*p* < 0.0001) and also greater than the IDH2R140 patients (*p* = 0.047). Moreover, 2,3-DHBA plasma concentrations were higher in the IDH2R172 patient than in either the IDH1R132 (*p* = 0.034) or IDH2R140 (*p* = 0.007) patients. It is concluded that the IDHR172 mutant neomorphic enzyme has a high activity with respect to the production of both 2R-HG and 2,3-DHBA. In addition, an analysis of six known mutant alleles [43,44] of the D-2-hydroxyglutarate dehydrogenase gene (*D2HGDH*) was conducted in WT patients by PCR. D2HGDH catalyzes the back reaction from 2R-HG to 2-OG [43]. Only one WT patient was found to have a *D2HGDH* mutation (A426T) but had unremarkable plasma concentrations of 2R-HG and 2,3-DHBA (see Tables S1 and S2). In addition, we examined *D2HGDH* mRNA expression in 46/51 patients with sufficient RNA. *D2HGDH* expression normalized to *ABL1* expression was unrelated to metabolite plasma concentrations of either 2R-HG or 2,3-DHBA (Figure S1) by Spearman rank correlation. These findings make it unlikely that D2HGDH activity, which mediates the conversion of 2R-HG back to 2-OG, has a dominant influence on plasma concentrations of either 2R-HG or 2,3-DHBA. This presumably remains due to mutated IDH.

The issue of the compartmentalization of 2R-HG and 2,3-DHBA was addressed because these metabolites are produced intracellularly but were determined in the plasma compartment. The expression of four transporter genes (*SLC16A1*, *SLC16A3*, *SLC22A6* and *SLC22A8*) was determined, normalized against *ABL1* expression. These were considered to be candidate transporters for the export of 2R-HG and 2,3-DHBA into plasma as both monocarboxylate transporters (MCT1 and MCT4) and organic anion transporters (OAT1 and OAT3). There was a weak and barely statistically significant negative concentration (*p* = 0.04) between both *SLC16A1* and *SLC16A3* expression and 2R-HG plasma concentration (Figure S2). The expression of these two transporters did not correlate with 2,3-DHBA plasma concentration. Neither *SLC22A6* or *SLC22A8* had detectable mRNA expression. The SLC16A1 and SLC16A3 findings are counterintuitive and may have no bearing on function because SLC16A1 (MCT1) is typically considered to be an importer from plasma and SLC16A3 (MCT4) is regarded as an exporter from cells to plasma [45]. 

#### 2.1.4. Biomarker Evaluation

It is to be expected that 2R-HG would act as a biomarker for mutations in *IDH1/2* as it is apparently only produced from 2-OG by the neomorphic IDH enzymes arising from mutated *IDH1/2* [7]. However, its utility as a biomarker for mutation of isocitrate dehydrogenase has not been evaluated in AML. Figure 2 clearly shows that there are AML patients without IDH1R132 and IDH2R140 mutations that have elevated plasma 2R-HG concentrations. These patients were assumed to be WT for *IDH1* and *IDH2*. It is possible that they bear other mutations. The known *IDH* mutations leading to the neomorphic enzyme all occur at the arginine residues IDH1R132, IDH2R140, and IDH2R172 [46]. Different mutations at these arginine residues have been reported to give rise to varying yields of 2R-HG over a nine-fold range in U87MG glioblastoma cells in culture, with IDH2R172M (Arg→Met) > IDH2R172K (Arg→Lys) > IDHR140Q (Arg→Glu). Cells expressing these mutated IDH enzymes have 2R-HG production determined by GC–MS that was 15- to 150-fold greater that cells expressing the WT enzyme [47]. We therefore examined the extent to which 2R-HG plasma concentration could act as a biomarker for *IDH1/2* gene mutation by using ROC analysis. In addition, if 2,3-DHBA is produced by IDH1R132 and IDH2140, then this metabolite should also be a biomarker for *IDH1/2* gene mutation when subjected to ROC analysis. As a negative control, plasma 3M2OVA concentrations were also utilized for ROC analysis.

**Figure 6:**
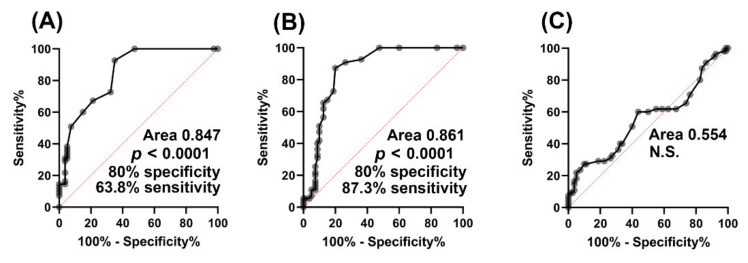
Receiver Operating Characteristic (ROC) curves for (**A**) plasma (2*R*)-hydroxyglutaric acid (2R-HG), (**B**) plasma (2*R*,3*S*)-dihydroxybutanoic acid (2,3-DHBA), and (**C**) (3*S*)-methyl-2-oxovaleric acid (3M2OVA) for mutant IDH1R132 and IDH2R140 vs. WT IDH1/2. Both plasma 2R-HG and plasma 2,3-DHBA highly statistically significantly (*p <* 0.0001) detected AML patients with *IDH* mutations, while plasma 3M2OVA did not. Note that at 80% specificity, plasma 2,3-DHBA had a better sensitivity (87.3%) than 2R-HG (63.8%).

The ROC analysis in Figure 6 further implicates mutant isocitrate dehydrogenase as the source of 2,3-DHBA in AML.

#### 2.1.5. Relationship of (2R)-Hydroxyglutaric Acid and (2R,3S)-Dihydroxybutanoic Acid to Clinical Variables of AML

Using multivariate analysis, we examined if the plasma concentrations of 2R-HG or 2,3-DHBA were related to prognostic risk (favorable, intermediate or adverse), plasma fibrinogen concentration, normal karyotype vs. aneuploidy, and FAB classification M0/M1/M2 vs. M4/M5/M6 at time of diagnosis (see Table S1). Plasma concentrations of 2R-HG were not related any of these variables associated with clinical outcome. However, plasma 2,3-DHBA was found to be 1.8-fold higher (*p* = 0.001) in patients with high plasma fibrinogen (≥4.1 g/L) relative to those with plasma fibrinogen concentrations in the normal range (<4.1 g/L). Patients with a plasma fibrinogen ≥4.1 g/L determined at the time of diagnosis have been reported to have poorer progression-free and overall survival [48]. This suggests that elevated plasma 2,3-DHBA, but not plasma 2R-HG, may be related to poorer survival. 

Multiple plasma metabolites, but not the oncometabolite 2R-HG, were found to be related to prognostic risk (favorable, intermediate or adverse), plasma fibrinogen concentration, normal karyotype vs. aneuploidy, and FAB classification M0/M1/M2 vs. M4/M5/M6 at time of diagnosis (Table S3). 

## 3. Discussion

Cancer metabolism is now a recognized hallmark of cancer [5]. The function of cellular metabolism in normal tissues is mainly concerned with the generation of energy through the breakdown of carbohydrates, fats, and amino acids and with the production of the building blocks such as phospholipids and nucleotides that are required for growth. In cancer cells, these metabolic processes are reprogrammed under multiple influences that include mutated oncogenes and tumor suppressor genes in order to support the proliferative cancer phenotype. The development of the field of metabolomics has permitted many novel insights into cancer metabolism, including oncogenic drivers of metabolic reprogramming [49], transcriptional regulators of metabolic rewiring in cancer cell lines [50], and abnormally expressed metabolic pathways in tumors [51]. 

In this investigation in AML patients, we have utilized plasma metabolomics to examine the metabolic consequences of *IDH1* and *IDH2* genotype determined from either peripheral blood mononuclear cells (PBMCs) or bone marrow mononuclear cells (BMMCs). As was to be expected, plasma 2R-HG concentration was greater in patients harboring mutations at either IDH1R132 or IDH2R140, since the neomorphic IDH enzyme they produce is responsible for synthesis of 2R-HG from 2-OG. Somewhat surprisingly, the difference between WT and mutated *IDH1/2* in plasma 2R-HG levels, although highly statistically significant (*p <* 0.0001), was determined to be only 7-fold. It is understood that 2R-HG is produced within leukemic cells that bear the *IDH1/2* mutations, which is a different compartment from the plasma that was subjected to metabolomic analysis. We did not collect either PBMCs or BMMCs for metabolomic analysis. It is probable that 2R-HG diffused or was transported from its site of production in leukemic cells into plasma. The expression of plasma membrane outward transporters in AML cells has not been characterized. The cellular export of 2R-HG might be expected to be elicited by the organic ion transporters 1 and 3 (OAT1 and OAT3, encoded by *SLC22A6* and *SLC22A8*, respectively), which mediate the cellular efflux of 2-OG [52], but the occurrence of these transporters in AML cells or their transport of 2R-HG have not been reported. Furthermore, the monocarboxylate transporters 1 and 4 (MCT1 and MCT4, encoded by *SLC16A1* and *SLC16A3*, respectively) that are typically overexpressed in glioblastomas, are underexpressed or silenced in glioblastomas that possess *IDH1* mutations. MCT1 and MCT4 typically uptake pyruvate and export lactate in tumor cells [53]. However, whether or not these transporters can move 2R-HG in or out of cells is currently unknown. Nevertheless, we examined the mRNA expression of four SLC transporter genes, namely*, SLC16A1, SLC16A3, SLC22A6* and *SLC22A8*. Neither of these last two transporters was expressed in the PBMCs studied. The weak negative and barely statistically significant correlation (*p* = 0.04) between SLC16A1 and SLC16A3 expression with 2R-HG plasma concentration did not explain the unexpectedly small difference between WT and mutant IDH1/2 in 2R-HG plasma concentration.

Next we examined if the mere 7-fold plasma concentration difference between *IDH1/2* mutated and WT AML patients was due to differential expression of D-2-hydroxyglutarate dehydrogenase (D2HGDH; EC 1.1.99.39), which catalyzes the reverse reaction of 2R-HG to 2-OG. There are known mutations in the *D2HGDH* gene leading to an inert enzyme. High expression of *D2HGDH* has also been found in head and neck squamous cell carcinoma (HNSCC), where it was reported to be a protective factor conferring low risk [54]. To date, the expression of *D2HGDH* in AML has not been reported, but as with HNSCC, it is possible that a high expression D2HGDH phenotype exists, which, if associated with IDH1/2, might explain the meager 7-fold difference in median plasma 2R-HG between mutated and WT IDH. In fact, Figure 2A shows considerable interpatient variability in plasma 2R-HG relative concentration in mutant *IDH1/2* patients with 40% of the WT patients but none of the mutant *IDH1/2* patients having values of zero or close to zero (≤ 0.1). For these reasons, we conducted the genotyping of six known inactivating *D2HGDH* alleles [43,44] in IDH1/2 WT AML patients. Only one inactivating mutation (A426T) was detected and in only one WT AML patient. However, this inactivating *D2HGDH* allele was associated with unremarkable plasma concentrations of both 2R-HG and 2,3-DHBA in that patient. Other as yet unreported *D2HGDH* inactivating alleles may explain why a few other IDH1/2 WT AML patients displayed elevated plasma concentrations of both 2R-HG and 2,3-DHBA. It should be noted that 2R-HG and its enantiomer 2S-HG are normal metabolites found in human body fluids [55] that are produced at low levels by mitochondrial alcohol dehydrogenase iron containing 1 (ADHFE1; EC 1.1.99.24) [56,57]. The mRNA expression of *D2HGDH* was then examined in 46/51 AML patients. No statistically significant correlations were found between *D2HGDH* expression and either 2R-HG or 2,3-DHBA plasma concentrations. It must be concluded that mutant IDH1/2 metabolic activity rather than D2HGDH is responsible for plasma levels of 2R-HG and 2,3-DHBA. 

The finding that plasma 2,3-DHBA levels were also highly statistically significantly (*p <* 0.0001) elevated in AML patients with *IDH1/2* mutations compared to WT (Figure 2B) was unexpected. Moreover, plasma levels of 2R-HG and 2,3-DHBA were highly statistically significantly correlated (*r_s_* = 0.569, *p <* 0.0001; Figure 3A). No correlation was found between 2R-HG and either glutamic acid (Figure 3B) or 3M2OVA (Figure 3C), which, like 2R-HG, can also be formed from 2-OG (Figure 4A). Finally, when examining WT and mutant *IDH1/2* using ROC analysis (Figure 6), 2,3-DHBA was better at discriminating mutated from WT *IDH1/2* than 2R-HG itself. These observations point to the production of 2,3-DHBA by mutant IDH1 and 2. The metabolic synthesis of 2,3-DHBA from its presumed precursor (3*S*)-hydroxy-2-oxobutanoic acid involves reduction of an α-keto (2-oxo) group to a (2*R*)-hydroxy group, as in the formation of 2R-HG from 2-OG (Figure 4). There is no known direct metabolic interchange between 2R-HG and 2,3-DHBA. In addition, there are relatively few citations for 2,3-DHBA. The presence of this metabolite in human urine has been documented on a limited number of occasions [38,40,58,59,60,61,62] and has also been described in the mouse [63] and a single dog with insulin-dependent diabetes mellitus (IDDM) [38]. The observation of abnormal threonine metabolism to 2,3-DHBA in both children and a dog with IDDM [38] raises the question as to whether or not our observations of elevated plasma 2,3-DHBA in AML with mutated *IDH1/2* could be due to diabetes. The relationship between diabetes mellitus and myelodysblastic syndromes (MDS) has been examined but there is no clear evidence that diabetes is a risk factor for MDS [64]. MDS can evolve into AML [65], but only seven of our 51 patients had a previous diagnosis of MDS (Table S1). It is unlikely therefore that IDDM with enhanced threonine catabolism [38] was an influence on elevated plasma 2,3-DHBA in AML patients with mutated *IDH1/2*. 

The principal implication of our findings is that neomorphic isocitrate dehydrogenase due to mutations at IDH1R132 and IDH2R140 not only reduces 2-OG to 2R-HG but also the threonine metabolite (3*S*)-hydroxy-2-oxobutanoic acid to 2,3-DHBA, two metabolic transformations that are remarkably similar. The biological characteristics of 2,3-DHBA are not known and whether or not this metabolite plays a role in AML pathogenesis is also undetermined. Therefore, whether or not 2,3-DHBA is an oncometabolite in AML remains to be determined. Moreover, regardless of any putative pathobiological properties of 2,3-DHBA, in the patient group described here, 2,3-DHBA appeared to be a better biomarker for *IDH1/2* mutations than even 2R-HG. Future research will need to confirm our findings in a larger genotyped cohort of AML patients and evaluate plasma 2,3-DHBA as a biomarker for *IDH1/2* mutation. The occurrence of 2,3-DHBA has mostly been reported in urine and therefore urinary 2,3-DHBA should also be evaluated for this biomarker. In addition, the biochemical conversion of (3*S*)-hydroxy-2-oxobutanoic acid into 2,3-DHBA requires confirmation in cell lines expressing IDH1 and IDH2 mutated enzymes. Furthermore, what role if any that 2,3-DHBA plays in modifying the epigenetic landscape of AML has not been investigated. Additionally, the pathogenetic properties of 2,3-DHBA will require investigation in AML and in other cancers where neomorphic IDH is known to play a role. Finally, and importantly, it remains to be established if knowledge of either 2,3-DHBA or 2R-HG plasma levels has clinical implications for the treatment of AML in patients in addition to *IDH1* or *IDH2* genotype. It is not the mutant genotypes per se that modify AML epigenetics, rather the oncometabolite 2R-HG. It has been established in this report that plasma levels of both 2R-HG and 2,3-DHBA are highly variable, both in WT and mutated *IDH1/2* patients. Therefore, plasma levels of these metabolites may have a significant bearing on the outcome and for the therapy of AML. Newly approved mutant IDH1 inhibitors such as ivosidenib can be used to treat relapsed or refractory IDH1-mutant AML [66]. The relapse of such patients after ivosidenib therapy has been reported to be due in part to new mutations occurring in the *IDH1* and *IDH2* genes [67]. This would be a circumstance where monitoring of plasma or urinary 2R-HG and 2,3-DHBA concentrations throughout therapy would provide a measure of drug efficacy that relies upon inhibition of mutant IDH1 and suppression of 2R-HG production. When plasma 2R-HG levels were monitored during a dose-ranging pharmacokinetic investigation of ivosidenib, 2R-HG levels were maximally and persistently inhibited in most, but not all, patients with advanced hematologic malignancies treated with 500 mg QD ivosidenib [68]. This demonstrates the value of such metabolic biomarkers in a precision medicine approach to drug treatment. In the same way, valuable insights into the progression of AML and its mechanisms could be obtained through the use of plasma 2R-HG and 2,3-DHBA as biomarkers.

## 4. Materials and Methods

### 4.1. Investigation of AML Patients

AML patients diagnosed and treated at the Inselspital University Hospital, Bern, Switzerland between 2009 and 2016 were included in this study. Informed consent of all patients in the study was obtained according to the Declaration of Helsinki, and the studies were approved by the ethics committee of Canton Bern, Switzerland (decision number #223/15). A total of 51 patients were recruited (30M, 21F) with a mean ± S.D. age of 62.8 ± 8.9 (range 40–82) years. Patient details are given in Table S1.

Routine mutational screening for myeloid panel genes including *FLT3, NPM1 and TP53* genes and conventional karyotype analysis of at least 20 metaphases were performed for each patient by the diagnostic department. Peripheral blood mononuclear cells (PBMCs) and bone marrow mononuclear cells (BMMCs) were collected at the time of diagnosis before the commencement of treatment. 

### 4.2. Genotyping IDH1,2 and D2HGDH

Genomic DNA was extracted from PBMCs or MBBCs. DNA fragments were amplified using FIREPol (Solis Biodyne) and gene-specific primers. For the *IDH1* gene, the primers were IDH1-F (5′- CCACCAACGACCAAGTCACCA-3’) and IDH1-R (5’-CAACATGACTTACTTGATCCCCA-3′). For the *IDH2* gene, the primers were IDH2-F (5′-CTCTGTCCTCACAGAGTTCAAGCT-3′) and IDH2-R (5′-GCCCGGTCTGCCACAAAGTCTGT-3′). For the *D2HGDH* gene, the primers were designed to cover the regions with the known mutations A208T, R212W, R421H, A426T, I147S, D375Y, N439D and V444A [43,44]. To cover A208T and R212W, the primers were F1 (5′-GGAGCTGAGCCGGTATGTGGAGGA-3′) and R1 (5′-GCCCATGAGAGCCGTGAGAGGACA-3′). R421H and A426T were covered with the primers F2 (5′-CTCGTCTCATCCTCTAGATGCT-3′) and R2 (5′-CTGCCCTGCCTCGAAGCCT-3′), I147S with F3 (5′-GGGGATGGTGGCGTAGGGGT-3′) and R3 (5′-CCCACAGTTGCAGACGCTGGCA-3′), D375Y with F4 (5′-CAGGCTCCAACGCAGGCCA-3′) and R4 (5′-CCCACACTGTCTAGGCTGCACCA-3′). N439D and V444A were covered with primers F5 (5′-GCCGGGGGTCTCGGGGT-3′) and R5 (5′-GCCCAGGACGTCCCTCTTCCT-3′). DNA sequences were analyzed by Sanger sequencing using the gene-specific primers.

### 4.3. Gene Expression Analysis Using Taqman Assays

RNA was extracted from mononuclear cells isolated from peripheral blood or bone marrow of AML patients and quantified using Taqman gene expression assays by Applied Biosystems, Thermo Fisher Scientific, Waltham, MA, USA. Total RNA was extracted using the Nucleospin RNAplus kit (740984.50, Macherey-Nagel, Düren, Germany). RNA was reverse transcribed using random primer and MMLV-RT (Promega, Madison, WI, USA). Relative quantitation was performed on the ABI7500 Real-Time PCR Instrument using FAST Start Universal probe master mix (Roche, Switzerland) and gene-specific Taqman probes provided by Thermofisher: Hs00292260_m1 (D2HGDH), Hs01560299_m1 (SLC16A1), Hs00358829_m1 (SLC16A3), Hs00537914_m1 (SLC22A6), Hs00188599_m1 (SLC22A8) and Hs01104728_m1 (ABL1). Measurements for D2HGDH, SLC16A1 and SLC16A3 were normalized with ABL1 values (ddCt relative quantitation). Assays were performed with four physical replicates each. 

### 4.4. GC–MS Metabolomics of AML Plasmas

EDTA anticoagulated plasma was frozen at −20 °C until analyzed. Thawed aliquots of all samples were pooled to create a series of quality control (QC) samples. Aliquots of plasma (50 µL) were subjected to GC–MS analysis in triplicate with 4-chlorophenylacetic acid (2 µM; 100 µL) as internal standard after the addition of ultrapure pyridine (200 µL; Merck, Darmstadt, Germany) and blown to dryness under N_2_ at 40 °C using a slight modification of our published methods [69,70]. Dried plasmas in capped glass tubes were then converted to their trimethylsilyl (TMS) derivatives with BSTFA containing 1% TMCS (250 µL; Sigma-Aldrich Chemie GmbH, Buchs, Switzerland) and ultrapure pyridine (100 µL) that was heated at 75 °C for 30 min, cooled and transferred to autoinjector vials. Derivatized plasmas were analyzed in batches of 30 that included a QC samples for the first five injections and then after every ten analytical samples. Samples (1.0 µL) were injected using an Agilent 7683B liquid sampler into an Agilent 6890N gas chromatograph with an Agilent 5975B mass selective detector operating under electron impact ionization at 70 eV. The front inlet was operated in splitless mode at 250 °C and an HP5-MS column (60 m; i.d. 250 µm; film thickness 0.25 µm) subjected to a temperature program of 70 °C for 3 min, 10 deg/min to 300 °C, held for 8 min (run time 34 min). Mass spectra were collected from m/z 35.0 to 650.0.

Raw chromatographic data yielded 67 chromatographic peaks that were annotated using the AutoQuant routine in MSD ChemStation (F 01.03.2357), employing the NIST 14 spectral library (MS Wil GmbH, Wil, Switzerland) that contained 276,248 mass spectra from 242,466 compounds. Confirmation of metabolite identities was made by comparison of their retention times and mass spectra with an in-house collection of 120 authentic standards. Six metabolites each appeared as two peaks due to multiple TMS derivatives and these concentrations were summed to yield relative concentrations (peak area ratio with the internal standard) for 60 identified metabolites. A spreadsheet of relative concentrations for each metabolite was constructed using Quant Browser GCMS software (Leoson BV, Middelburg, The Netherlands) [69,70], which was then imported into SIMCA 16 (Sartorius Stedim, Goettingen, Germany) for multivariate data analysis. 

### 4.5. Multivariate Data Analysis and Univariate Statistics

Datasets (WT IDH1/2, IDH1R132, IDH2R140, and QC) were first analyzed by multivariate data analysis using unsupervised principal components analysis (PCA) to examine the internal structure of the datasets and the occurrence of any outliers in the PCA scores plots [32]. Next, data were assigned to classes (WT IDH1/2, IDH1R132, IDH2R140, and QC) and subjected to supervised analysis using partial least squares projection to latent structures-discriminant analysis (PLS-DA) to test for clustering and segregation of the different datasets in PLS-DA scores plots [32]. The PLS-DA models were tested for potential overmodeling by a cross-validation procedure that repetitively (200 iterations) removed 1/7 of the data, randomized the remaining 6/7 and returned the 1/7 previously removed. The R2 (correlation) and Q2 (predictability) coefficients should degrade to <0.3 and <0, respectively, if the data are not overmodeled [70]. After validating the PLS-DA models, data were finally subjected to orthogonal PLS-DA (OPLS-DA), where the loadings can be expressed in the form of an S-plot that shows metabolites that are up- and down-regulated in different datasets [70,71,72,73]. Metabolites selected by OPLS-DA were then subjected to univariate statistics using GraphPad Prism 8.4.3. All statistical determinations were nonparametric to allow for non-Gaussian distribution of data. Specifically, for comparison between groups, a two-tailed Mann–Whitney test was used to compare median values with *p*-value cut offs for statistical significance lowered below *p* = 0.05 by use of the Bonferroni correction for multiple comparisons [74]. For correlations between datasets, Spearman rank correlation was used. Receiver operating characteristic (ROC) curves were also generated using Prism.

Raw GC–MS data are available at http://doi.org/10.6084/m9.figshare.13014278.

## 5. Conclusions

It is concluded that mutant isocitrate dehydrogenase IDH1R132 and IDH2R140 in AML patients not only metabolically reduces 2-oxoglutaric acid to the oncometabolite (2*R*)-hydroxyglutaric acid (2R-HG) but also elicits the reduction of the threonine metabolite (3*S*)-hydroxy-2-oxobutanoic acid to (2*R*,3*S*)-dihydroxybutanoic acid (2,3-DHBA). The products of both reactions bear chemical and stereochemical similarities. The two metabolic products were highly statistically significantly elevated in the plasma of AML patients with IDH1R132 and IDH2R140 mutations compared to patients with wild-type IDH1/2. These two plasma metabolites were strongly correlated across all patients studied. D-2-Hydroxyglutarate dehydrogenase (*D2HGDH*) inactivating alleles are unlikely to explain elevated both 2R-HG and 2,3-DHBA plasma concentrations in AML patients that are WT for IDH1/2 since the expression of *D2HGDH* did not correlate with 2R-HG and 2,3-DHBA plasma concentrations. ROC analysis established that 2,3-DHBA was a better biomarker for *IDH1/2* mutation in AML than 2R-HG. Metabolomic analysis revealed major differences in the plasma metabolome between WT IDH1/2, IDH1R132 and IDH2R140 patients, with 21 metabolites displaying statistically significant differential plasma concentrations in the order IDH2R140 > WT IDH1/2 > IDH1R132. These were mostly α-amino acids and urea cycle intermediates, suggesting differential effects of these two mutations on AML biology. In addition to a potential role as a plasma biomarker for *IDH1/2* mutation in AML, this little studied metabolite 2,3-DHBA may play an etiological role in leukemogenesis. Plasma 2R-HG and 2,3-DHBA as biomarkers may assist in monitoring drug therapy for AML and enhance our understanding of disease progression and relapse.

## Figures and Tables

**Table 1:** Plasma metabolite relative concentration (metabolite peak area/internal standard peak area) differences between wild-type AML patients and those with IDH1R132 and IDH2R140 mutations. The three groups were compared using nonparametric ANOVA (Kruskal–Wallis test) with *p*-values given.

#	Metabolite	Median Relative Concentration Wild-Type IDH1/2	Median Relative Concentration IDH1R132	Median Relative Concentration IDH2R140	*p*-Value
1	galactose	541	372	595	<0.0001
2	mannose	265	170	302	<0.0001
3	xylitol	0.6	0.3	0.8	<0.0001
4	alanine	14.7	7.7	23.5	<0.0001
5	valine	32.4	26.4	47.6	<0.0001
6	leucine	29.1	23.8	38.4	<0.0001
7	isoleucine	10.2	7.9	14.0	<0.0001
8	proline	17.2	9.3	20.3	<0.0001
9	tyrosine	20.2	14.4	26.9	<0.0001
10	glutamine	8.2	3.7	9.2	<0.0001
11	phenylalanine	11.5	9.4	12.5	<0.0001
12	tryptophan	12	8.6	16.5	0.0002
13	methionine	1.8	1.2	2.2	<0.0001
14	lysine	0.6	0.3	1.1	<0.0001
15	glutamic acid	0.8	0.5	1.2	<0.0001
16	ornithine	0.2	0.1	0.4	<0.0001
17	ethanolamine	14.5	9.2	20.2	<0.0001
18	carbamic acid ^1^	3.6	3.1	3.8	<0.0001
19	threonine	6.2	4.0	7.7	0.0001
20	hexanoic acid	0.8	0.8	0.7	0.001
21	urea	965.4	285	1051	0.02
22	serine	7.2	3.9	8.9	<0.0001

^1^ Formed from carbamoyl phosphate by thermal decomposition [33].

## Supplementary Materials

The following are available online at https://www.mdpi.com/2072-6694/12/10/2842/s1, Figure S1: Lack of correlations between *D2HGDH* mRNA expression 2R-HG and 2,3-DHBA plasma concentration, Figure S2: Correlations between *SLC16A1* and *SLC16A3* mRNA expression and 2R-HG and 2,3-DHBA plasma concentration, Table S1: Patient characteristics, Table S2: Triplicate plasma relative concentrations of 60 metabolites for each AML patient, Table S3: AML clinical variables and effect on the plasma metabolome.
