# Supplementary material for: (2R,3S)-Dihydroxybutanoic Acid Synthesis as a Novel Metabolic Function of Mutant Isocitrate Dehydrogenase 1 and 2 in Acute Myeloid Leukemia
Source: Cancers (Basel). 2020 Oct 1;12(10):2842. doi: 10.3390/cancers12102842 (PMC7600928; doi:10.3390/cancers12102842)
Supplement: Supplementary file 1 [file cancers-12-02842-s001.zip › Figure S2.pdf]

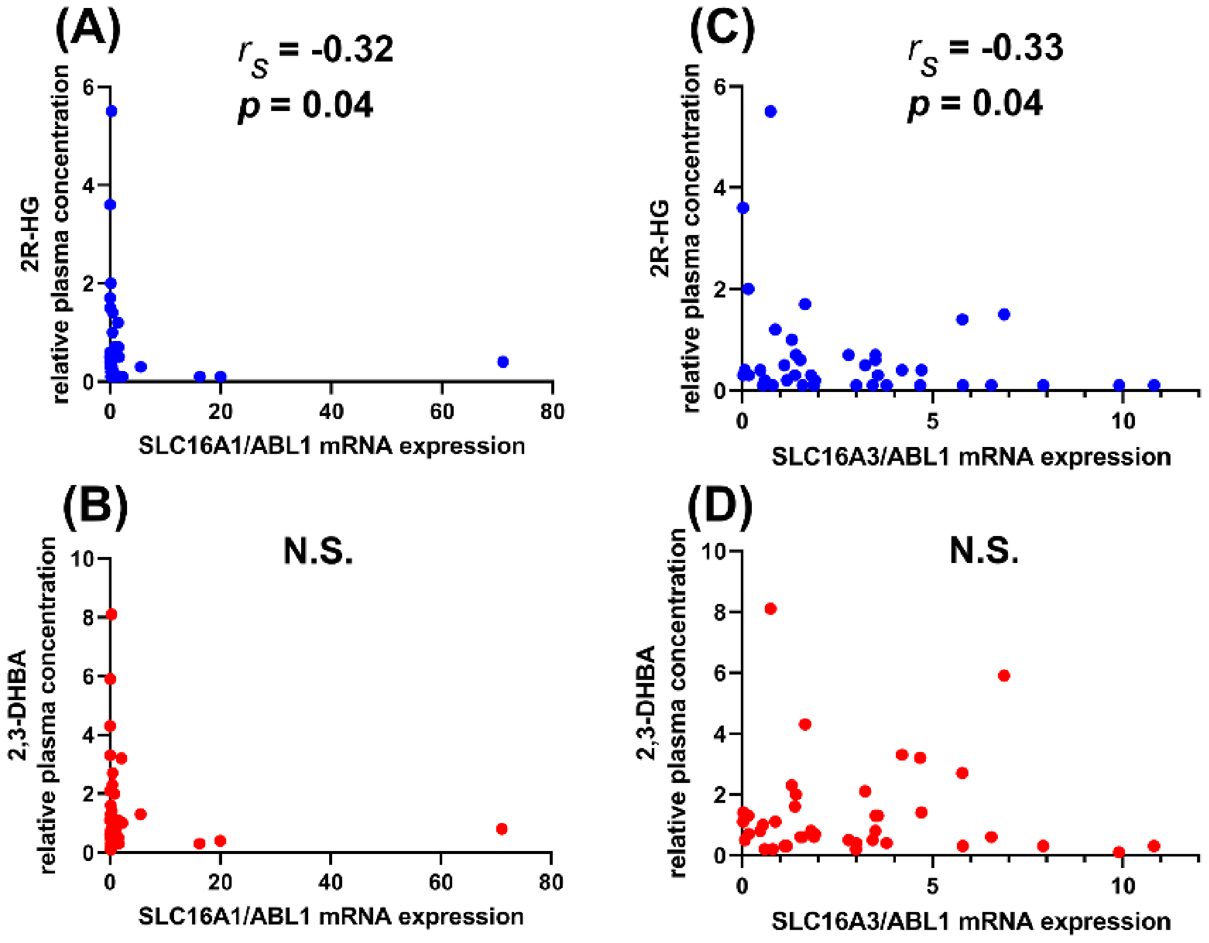

Figure S2. (A) Weak negative correlation (r_S = -0.32; p = 0.04) between *SLC16A1* mRNA expression (normalized to *ABL1*) and 2R-HG plasma concentration; (B) absence of a correlation between *SLC16A1* mRNA expression (normalized to *ABL1*) and 2,3-DHBA plasma concentration; (C) Weak negative correlation (r_S = -0.33; p = 0.04) between *SLC16A3* mRNA expression (normalized to *ABL1*) and 2R-HG plasma concentration; (D) absence of a correlation between *SLC16A3* mRNA expression (normalized to *ABL1*) and 2,3-DHBA plasma concentration
