# Supplementary material for: (2R,3S)-Dihydroxybutanoic Acid Synthesis as a Novel Metabolic Function of Mutant Isocitrate Dehydrogenase 1 and 2 in Acute Myeloid Leukemia
Source: Cancers (Basel). 2020 Oct 1;12(10):2842. doi: 10.3390/cancers12102842 (PMC7600928; doi:10.3390/cancers12102842)
Supplement: Supplementary file 1 [file cancers-12-02842-s001.zip › Table S3.pdf]

Table S3. AML clinical variables and effect on the plasma metabolome

Favorable vs Adverse Prognostic Risk

metabolite	median relative concentration Favorable	median relative concentration Adverse	fold-change (Favorable/ Adverse)	P
inorganic phosphate	0.0958	0.0634	1.5	<0.0001
dodecanoic acid (12:0)	0.0014	0.0010	1.4	<0.0001
myristic acid (14:0)	0.0057	0.0043	1.3	<0.0001
9-hexadecenoic acid (16:1)	0.0051	0.0035	1.5	0.002
inorganic pyrophosphate	0.0049	0.0040	1.2	0.002
palmitic acid (16:0)	0.0725	0.0563	1.3	0.004

Favorable vs Intermediate Prognostic Risk

metabolite	median relative concentration Favorable	median relative concentration Intermediate	fold-change (Favorable/ Intermediate)	P
tyrosine	0.0177	0.0225	-1.3	<0.0001
tryptophan	0.0104	0.0157	-1.5	<0.0001
proline	0.0147	0.0187	-1.3	0.004
valine	0.0318	0.0386	-1.2	0.01
alanine	0.0134	0.0203	-1.5	0.01

Low (<4.1 g/L) vs High (≥4.1 g/L) Fibrinogen

Metabolite	median relative concentration Low fibrinogen	median relative concentration High fibrinogen	fold-change (Low/High)	P
galactose	0.5799	0.3719	1.6	<0.001
(2*R*,3*S*)-dihydroxybutyric acid	0.0011	0.0020	-1.8	0.001
carbamic acid	0.0036	0.0031	1.2	0.002
hexanoic acid	0.0007	0.0009	-1.3	0.002
1-palmitoylglycerol	0.0094	0.0113	1.2	0.003

Normal vs Aneuploidy

Metabolite	median relative concentration Normal	median relative concentration Aneuploidy	fold-change (Normal/ Aneuploidy)	P
dodecanoic acid (12:0)	0.0013	0.0010	1.3	<0.0001
1-oleoylglycerol	0.0002	0.0003	-1.5	0.0002
9-hexadecenoic acid (16:1)	0.0049	0.0032	1.5	0.0002
hypoxanthine	0.0011	0.0006	1.8	0.0006
decanoic acid (10:0)	0.0005	0.0003	1.7	0.002
glucose	0.2929	0.3691	-1.3	0.003

inorganic phosphate	0.0873	0.0695	1.3	0.003
myristic acid (14:0)	0.0050	0.0042	1.3	0.004
**FAB classification M0/M1/M2 vs M4/M5/M6**				
Metabolite	median relative concentration M0/M1/M2	median relative concentration M4/M5/M6	fold-change (M0/M1/M2/ M4/M5/M6)	P
α-tocopherol	0.0064	0.0051	1.3	<0.0001
hypoxanthine	0.0006	0.0013	-2.2	0.0001
succinic acid	0.0007	0.0012	-1.7	0.001
cholesterol	0.0716	0.0657	1.2	0.006

Relative concentration refers to the ratio between the area of the metabolite peak and the area of the internal standard (4-chlorophenylacetic acid) peak.

Positive and negative values of fold-change indicate first data column/second data column ratios and second data column/first data column ratios, respectively.

Statistical significance of metabolites was subjected to Bonferroni correction for multiple comparisons.
